# How different contexts of social capital are associated with self-rated health among Lithuanian high-school students

**DOI:** 10.1080/16549716.2018.1477470

**Published:** 2018-06-06

**Authors:** Dario Novak, Arunas Emeljanovas, Brigita Mieziene, Lovro Štefan, Ichiro Kawachi

**Affiliations:** a Faculty of Kinesiology, University of Zagreb, Zagreb, Croatia; b Department of Health, Physical and Social Education, Lithuanian Sports University, Kaunas, Lithuania; c Department of Social and Behavioral Sciences, Harvard T.H. Chan School of Public Health, Boston, Massachusetts, USA

**Keywords:** Social capital, adolescents, family support, school support, vertical trust, horizontal trust

## Abstract

**Background**. Adolescents’ self-rated health is related to a number of sociodemographic and socio-economic factors, health-related behaviors, and their social environment. The impact of the latter is still not well explored. An adolescent’s social environment is represented by the social capital, i.e. social resources that they can access. The relationships between various contexts of social capital (family, neighborhood, peers, and school) and self-rated health among adolescents are still unclear.

**Objective**. This study aims to examine the relationships between various social capital contexts and self-rated health in Lithuanian adolescents.

**Methods**. The current cross-sectional study includes a nationally representative sample of 1863 adolescents (51.4% were girls) aged 14–18 years. The indicators of self-rated health as well as indicators of social capital in family, neighborhood, and school contexts were assessed. The results of the relationships between self-rated health and contexts of social capital were calculated controlling for the following covariates: physical activity, psychological distress, gender, body mass index, and family socioeconomic status.

**Results**. Results indicate that there are significant relationships between good self-rated health and a higher level of family support, neighborhood trust, and vertical school trust. In the final logistic regression model, while controlling for all covariates, a higher level of family support and neighborhood trust remain significant predictors of good self-rated health.

**Conclusions**. Family support and neighborhood trust are important correlates of self-rated health in adolescents.

## Background

Self-rated health in adolescence is an important predictor of health in adulthood [1]. Self-rated health is a subjective, change-sensitive measure of health which covers the ability to function in physical, social, and psychological contexts [2,3]. Adolescents’ self-rated health is related to a number of factors, beginning with sociodemographic factors, such as age, gender, family, and socioeconomic status [4–7]; followed by health-related behavior, such as mental health [7]; and ending with social environment [6].

A social network in adolescence covers mostly the contexts of family, school, peers, and neighborhood [8]. Research results have raised concerns about the quality of the social network of Lithuanian adolescents. In Lithuania, from 42% to 72% of girls and boys find it easy to communicate with their parents. From 18% to 26% of girls and boys have less than three friends [9]. Only, about 40% of girls and 30% of boys like school [9]. Just a little over half (55%) of 15-year-old adolescents trust their classmates. Lithuania has, as have many other Eastern Europe countries, undergone a political and societal transition since 1990. These changes affected family life through reduced birth rates, higher divorce rates, changes in family status and structure, and so on [10]. Education in Lithuania is free and compulsory at the primary and basic educational level (from 6 or 7 years of age up to 16 years of age), as stated in the National Law on Education. Adolescents can choose an upper secondary education at either a high school or vocational school. Also, they are able not to choose any further education. Boys and girls study together at all levels.

Some authors report that lower rates of peer support are associated with depression [11] and a higher prevalence of substance abuse [12]. The school environment has a potential benefit for adolescents’ self-esteem, behavior, and future life-satisfaction [13]. So, the quality of social relationships in adolescence is very important as it has been confirmed that youth who are close to their parents report higher self-rated health [2] as well as fewer physical and psychological problems [14]. Close friendship ties represent a critical development task in young people, affecting their social adjustment [15] and competencies [16].

These social networks based on family, peers, school, and neighborhood are the grounds for social capital for adolescents. Social capital is a measure of particularized and generalized trust, social participation, integrity and norms of reciprocity [17]. It also represents resources accessed through social networks [18] that help people to achieve their goals, facilitate coordination, communication, resolution of collective dilemmas, reduce the incentives for opportunism, broaden the participants’ sense of self, developing perception of ‘we’ [19–22]. The distinction is made between bonding social capital, which results from interactions within a group of people like oneself, and bridging as well as linking social capital, both of them represent interactions with people from different groups. While bridging social capital refers to horizontal trust among different groups at the same level of social scale, linking social capital describes relationships among groups with power differences (e.g. patron/client or mentor/mentee relationships) [22]. This also suggests the multi-contextual nature of social capital, identifying the different social networks that contribute to securing adolescents’ sense of belonging. Other studies suggest that, by assessing the relative importance of adolescents’ social capital within the family, school, and neighborhood contexts, a better understanding of the impact of these contexts on adolescents’ self-rated health will be obtained [23].

However, the relationship between social capital and health in adolescence remains interesting [24,25] and still understudied. Most of the previous studies investigated the relationships between social capital and self-rated health in adults [26,27], leaving these relationships unclear in adolescents. Explorations of the relationships between social capital and self-rated health among high-school students are still scarce [28,29].

It is important to investigate possible associations between social capital and self-rated health in different countries due to their different socio-economic statuses, cultures, and educational systems. This could lead to a more focused approach directed toward a specific country instead of broad and general health interventions being adopted based on social capital. Thus, the current study aims to examine the relationships between various social capital contexts and self-rated health in Lithuanian adolescents.

## Methods

### Study design and sample

This cross-sectional study was performed on a nationally representative sample of Lithuanian adolescents. The study was carried out across all 10 regions of Lithuania. Cluster (Area) random sampling was used. Urban and rural areas were proportionally represented. Within each region, two schools (primary sampling units) were selected: one from the main city and one from the district. School codes were written on slips of paper and placed inside a box. Schools were randomly selected by choosing school codes from the box, with each school code having an equal probability of selection. Twenty schools, in total, were selected. Six schools refused to participate in the study. One of each of the 9th, 10th, 11th and 12th grades or 1st – 4th gymnasium classes (secondary sampling units) were chosen at each school. Both the school and class in the school were considered to be clusters. All the children within the sampled clusters were measured.

Informed consent from parents and adolescents was obtained. Students completed the anonymous paper questionnaires in the classroom with the researchers present. The aim and procedures of the study were explained before the questionnaires were completed. Completing the questionnaires took approximately 30 minutes. The researchers helped students, if needed.

### Participants

A total of 1863 adolescents (906 boys and 957 girls) of 14–18 years of age were included in the study during the 2015–2016 school year. Of all the included students, 15 (0.8%) did not wish to participate, while 50 (2.7%) students returned the questionnaires with incomplete data.

### Measurements

#### Self-rated health

Participants were provided the question ‘How would you estimate your health?’ to assess their perceived health. Responses were distributed on a five-point Likert-scale, ranging from very poor (1), poor (2), average (3), good (4), to excellent (5). Responses ‘very poor’ and ‘poor’ were categorized as ‘poor,’ and responses ‘fair,’‘good,’ and ‘excellent’ were categorized as ‘good’ self-rated health [30]. Perceived health, in turn, is related to many aspects of physical and mental health and has been used as a measure to predict mortality in adults [31,32] and adolescents [33,34].

#### Social capital

Adolescents’ social capital was assessed in family, neighborhood, and school contexts [35]. Social capital in the context of family, representing family support, was assessed using one question: ‘Do you feel that your family understands and cares about you?’ Social capital in the neighborhood context was assessed using two questions: ‘Do you feel people trust each other in your neighborhood?’ (neighborhood trust), and ‘Do you feel that your neighbors step in to criticize if they see a high-school youth engaged in antisocial behavior?’ (informal social control). Social capital in the context of school was assesses via three questions: ‘Do you feel that teachers and students trust each other in your high school?’ (vertical school trust), ‘Do you feel students trust each other in your high school?’ (horizontal trust), and ‘Do you think students collaborate with each other in your high school?’ (reciprocity). The responses were indicated on a five-point Likert-scale: (1) strongly agree, (2) agree, (3) neither agree nor disagree, (4) disagree, and (5) strongly disagree. The answers were binarised as ‘high’ (strongly agree and agree) and ‘low’ (neither agree nor disagree, disagree, and strongly disagree) social capital [35].

### Covariates

The short version of the International Physical Activity Questionnaire (IPAQ) was used to assess physical activity. The indicator expressed the metabolic equivalent of hours per week [36]. Participants were categorized as highly active (60 min or more per day), medium active (30–59 min per day) and low active (<30 min per day) [37]. Body mass index was calculated from self-reported height and weight. The indicator discriminated between respondents being and not being overweight or obese (scoring of responses in the range ≥25 kg/m^2^vs <25 kg/m^2^). Socio-economic status was based on both parents’ occupations at the time that the research was conducted and recorded as high (i.e. managers and professionals), middle (white- collar), and low (blue-collar) socio-economic status. This category was further dichotomized as high/middle (responses in the range 1–4) and low (responses in the range 5–6) [30,38]. Psychological distress was assessed using the six-item Kessler scale [39]. Each question (e.g. ‘“How often during the past 30 days did you feel nervous?”’) was scored from 0 (none of the time) to 4 (all of the time). Item scores were summed up (0–24), with a lower score indicating a lower level of psychological distress. The Cronbach α was .879, meaning that the scale showed good internal consistency. Following previous research, the summed score was dichotomized into two categories, indicating low (0–12 points) and high (>13) psychological distress [39].

### Statistical analysis

Data were analyzed using SPSS 18.0 software (SPSS Inc. Chicago, IL USA). Descriptive statistics were used to determine the percentage distribution of answered questions. The Chi-square test was used to identify differences between categorical variables. The relationships between social capital contexts and self-rated health were identified using multivariate logistic regression, controlling for the covariates gender, body mass index, family socio-economic status, psychological distress and physical activity. Four models were examined: the relationship between family support and self-rated health (Model 1), neighborhood social trust and self-rated health (Model 2), school social trust and self-rated health (Model 3), and all social capital determinants and self-rated health (Model 4). Statistical significance was set at a p-value of less than 0.05.

## Results

Preliminary analysis (Table 1) and comparison among genders revealed that, overall, 43.6% of all participants – 34.1% of boys and 52.7% of girls – reported poor self-rated health. The results indicate that girls are significantly more dissatisfied with their health than boys. Most of the students had normal body mass index. Girls were less likely to be overweight or obese than boys. Differences between genders did not occur when reporting socio-economic status. Almost 78% of participants reported low socio-economic status. Girls reported high psychological distress more often (31.4%) than boys (12.5%). Among all participants, 77.1% reported doing high/moderate physical activity. Boys were active more often than girls. Family support and reciprocity at school did not differ between genders. While boys showed higher neighborhood trust and horizontal school trust than girls, girls showed higher vertical school trust than boys.

**Table 1.** Characteristics of the high-school students, participants to the Social capital survey, Lithuania, 2016.

	Boys(N = 906)	Girls (N = 957)
	N (%)	N (%)
**Self-rated health**		
Poor	309 (34.1)	504 (52.7)
Good	597 (65.9)	453 (47.3)
**Family social capital**		
Low	110 (12.1)	116 (12.1)
High	796 (87.9)	841 (87.9)
**Neighborhood trust**		
Low	407 (44.9)	537 (56.1)
High	499 (55.1)	420 (43.9)
**Informal social control**		
Low	652 (72.0)	639 (66.8)
High	254 (28.0)	318 (33.2)
**Vertical school trust**		
Low	419 (46.2)	507 (53.0)
High	487 (53.8)	450 (47.0)
**Horizontal school trust**		
Low	360 (39.7)	499 (52.1)
High	546 (60.3)	458 (47.8)
**Reciprocity at school**		
Low	207 (22.8)	224 (23.4)
High	699 (77.2)	733 (76.6)
**Body mass index**		
Normal	803 (88.6)	893 (93.3)
Overweight/obese	103 (11.4)	64 (6.7)
**Self-perceived socioeconomic status**		
High/middle	206 (22.7)	209 (21.8)
Low	700 (77.3)	748 (78.2)
**Psychological distress**		
High	113 (12.5)	301 (31.4)
Low	793 (87.5)	656 (68.6)
**Physical activity**		
High/moderate	746 (82.3)	266 (27.8)
Low	160 (17.7)	691 (72.2)

The relationships between social capital contexts and self-rated health are presented in Table 2. Results show that good self-rated health was significantly related to higher levels of: family social support (OR 1.83; 95% CI 1.35 to 2.48; p < .001), neighborhood trust (OR 1.44; 95% CI 1.19 to 1.75; p < .001), and vertical school trust (OR 1.35; 95% CI 1.05 to 1.63; p < .05). When controlled for all covariates, good self-rated health was significantly related to a higher level of family social support (OR 1.66; 95% CI 1.22 to 2.56; p < .001) and neighborhood trust (OR 1.29; 95% CI 1.05 to 1.58; p < .05). Girls, overweight/obese, physically inactive, and low family socio-economic status adolescents were less likely to report good self-rated health, except in the case of self-perceived socio-economic status in Model 4 (p > .05). Those who had a low level of psychological distress were more likely to report good self-rated health.

**Table 2.** The association* between social capital determinants and good self-rated health in high-school students, Lithuania, Social capital survey, 2016.

	Model 1OR (95% CI)	Model 2OR (95% CI)	Model 3OR (95% CI)	Model 4OR (95% CI)
**Family social capital**				
Low				
High	1.83*** (1.35–2.48)			1.66*** (1.22–2.56)
**Neighborhood trust**				
Low				
High		1.44*** (1.19–1.75)		1.29* (1.05–1.58)
**Informal social control**				
Low				
High		0.96 (0.77–1.18)		0.93 (0.76–1.15)
**Vertical school trust**				
Low				
High			1.31* (1.05–1.63)	1.23* (1.00–1.54)
**Horizontal school trust**				
Low				
High			1.15 (0.92–1.44)	1.10 (0.87–1.39)
**Reciprocity at school**				
Low				
High			1.03 (0.80–1.32)	0.97 (0.76–1.25)
**Gender**				
Boys				
Girls	0.52 (0.43–0.64)***	0.55 (0.45–0.67)***	0.54 (0.45–0.66)***	0.54 (0.44–0.66)***
**Body mass index**				
Normal				
Overweight/obese	0.50 (0.36–0.71)***	0.51 (0.36–0.72)***	0.50 (0.35–0.70)***	0.50 (0.35–0.70)***
**Self-perceived socioeconomic status**				
High/middle				
Low	0.79 (0.63–0.99)*	0.77 (0.62–0.98)*	0.78 (0.62–0.98)*	0.81 (0.64–1.02)
**Psychological distress**				
High				
Low	0.49 (0.38–0.62)***	0.48 (0.38–0.61)***	0.49 (0.38–0.62)***	0.53 (0.41–0.68)***
**Physical activity**				
High/moderate				
Low	0.54 (0.43–0.68)***	0.54 (0.43–0.68)***	0.54 (0.43–0.68)***	0.55 (0.44–0.69)***

*: association measured as Odds Ratio from logistic regression model

**Model 1**: examine the associations between family social capital and youth self-rated health adjusting for gender, body mass index, self-perceived socioeconomic status, psychological distress and physical activity.

**Model 2**: examine the associations between neighborhood social capital and youth self-rated health adjusting for gender, body mass index, self-perceived socioeconomic status, psychological distress and physical activity.

**Model 3**: examine the associations between school social capital and youth self-rated health adjusting for gender, body mass index, self-perceived socioeconomic status, psychological distress and physical activity.

**Model 4**: examine the associations between all social capital variables and youth self-rated health adjusting for gender, body mass index, self-perceived socioeconomic status, psychological distress and physical activity.

*p < 0.05; **p < 0.01; ***p < 0.001; OR odds ratio; CI confidence interval

## Discussion

The current study was aimed at examining the relationships between various social capital contexts and self-rated health in Lithuanian adolescents. This relationship is very important, as studies show that poor self-rated health at a young age is related to increased risk of mortality later in life [40].

Results in the current study have shown a strong relationship between self-rated health and family support. These results are similar to the results from other studies [2,30]. Family support undoubtedly represents a key element for children’s health, as a perception of belonging to the family is related with health behaviors in childhood [41]. Also, after achieving independence in the 1990s, Lithuania moved from socialism to capitalism. Due to the resulting rapid socioeconomic development, the family, in particular, became the main source of social and financial support for children [28].

A healthy community is also an important factor in the development of health in children. Our results have shown a strong relationship between neighborhood trust and self-rated health. These are similar to the results obtained in a study conducted by Novak et al. [29]. The authors reported that those adolescents who live in highly trusted communities report better self-rated health than those whose neighborhood is poorly trusted [29]. Khawaya and colleagues [28] investigated the associations between social capital and self-rated health for adolescents 13–19 years old in Beirut. These authors found that distrust was more widespread among adolescents reported to be living in poor suburban communities. In these poor suburban communities, adolescents with lower social capital were four times more likely to report poor self-rated health than those who had higher social capital. Neighborhood social capital had a direct effect on youth’s health, where adolescents with higher reported self-rated health accessed formal (hospitals) and informal (family, friends) healthcare systems at higher rates [42].

This study did not show a significant relationship between informal social control and self-rated health among high-school students. These results are similar to those found by a study of Croatian adolescents, in which informal social control did not seem to affect self-rated health [30]. Compared to the current study results, Drukker and colleagues [43] determined that informal social control was related to self-rated health. Youths living in neighborhoods high in social capital showed better general health, mental health, behavior, and satisfaction [43]. Moreover, a high level of informal social control could prevent delinquent behavior, with higher level of mutual protection [44]. The absence of a relationship between informal social control and self-rated health in Lithuanian adolescents might be explained by the fact that more than two thirds of the Lithuanian adolescents perceive it as low, and by the assumptions that adolescents probably did not consider neighbors criticism of youth’s antisocial behavior as a positive issue or they care about it not that much to affect their perceptions of health. Since Lithuania was formerly part of the Eastern bloc and under the influence of the ex-Soviet Union, where social responsibility was not widespread and criticism in the neighborhoods was not also welcomed, it is possible that the former totalitarian regime is still affecting parents and, as a consequence, their children.

School social capital was not significantly related to self-rated health, with the exception of vertical school trust in Models 3 and 4, identifying that adolescents who perceived high vertical trust were more likely to report good self-rated health. These results remained significant even after adjusting for gender, body mass index, self-rated economic status, psychological distress and physical activity. So, resources and support received outside adolescents’ own social networks are of particular importance determining their perception of own health status. These positive effects of vertical trust for perceived health in adolescents might be developed through health promotion and healthy norms spread and adopted, and social control over deviant behavior [45]. Research show that vertical trust, which reflects linking social capital, was also associated with self-rated health in other populations. Higher vertical trust was related to better oral self-rated health in Japanese college students [36], higher linking social capital was related to higher levels of health in both urban and rural areas of China [46]. On the contrary, low linking social capital associated with higher risk of poor self-rated health in Swedish adults [47]. Ferlander suggests that linking social capital is important for health in terms of the control of deviancy and reinforcement of positive health norms in society [48].

The absence of a relationship between horizontal school trust and reciprocity at school and self-rated health in the final model (Model 4) might be explained by a study which found that schoolchildren do not like going to school and do not find their classmates to be kind and helpful [9]. Also, roughly 50% of our participants reported low horizontal and vertical school trust, most likely pointing to social interaction between adolescents in schools being based on competitive behaviors between individuals and groups. It should also be indicated that each indicator of social capital in this study is based on adolescents’ perceptions about the social context. Any fact-level information was gathered within social capital contexts. Still, these individual perceptions reflect a personal sense of belonging, which could be more a important predictor of other self-perceived factors than facts, themselves.

The control variables – gender, body mass index, psychological distress, and physical activity which were included in the logistic regression equations and all were significant for self-rated health. Being female, overweight or obese, having a low rate of physical activity, or having a high rate of psychological distress doubles the chances of having lower self-rated health. These results are similar to other studies. In line with other research, girls consistently rated their health lower than boys. A large international study on adolescents’ health-related factors revealed that girls have a poorer perception of their health in comparison to boys, at all ages, and in all 29 countries surveyed [4]. Vingiliset al. [49] found that youth 12–19 years of age in the top quintile (20%) of body mass index had lower health ratings. On the other hand, their study discovered the interesting result that youth who were in the highest tertile of physical activity had lower self-rated health. Hence, scientific evidence on the link between physical activity and self-rated health is contradictory. Higher psychological distress is related to lower self-rated health in the study of Vingilis and colleagues [49], which is in line with our results.

In the future, an action plan needs to be incorporated into the educational system for tracking how social capital develops between children, and between children and their families and neighbors. For example, sports are often regarded as one of the key contributors to building positive social capital [50]. Also, it is important that policy makers, and, particularly in this case, Department of Education officers and school principals, be aware of social capital implications on adolescents’ health and life. This way these individuals could propose and incorporate actions that could foster the social capital stock among this population. Research has also demonstrated that, when building social capital in youth, the information, support, and encouragement provided by adults are the resources that are most required. So, organized youth programs aimed at developing these resources are necessary [51].

Our study has several limitations. First, we have not examined the difference among adolescents living in single- vs. two-parent families. Living with both parents may increase a child’s perception of safety, protection, and wellbeing, among other factors. Second, keeping in mind the cross-sectional design of the study, reverse causality might also be taking place, i.e. self-rated health could be a predictor of family support and the other determinants included in the model. To reduce this risk, results were adjusted for psychological distress. This was based on the premise that adolescents having higher psychological distress are expected to report lower levels of social capital. In addition, psychological distress could potentially affect self-ratings of health. Therefore, individual differences in psychological distress were adjusted in order to eliminate this possible bias. Third, unfortunately, the survey did not included information about structural social capital indicators (e.g. participation). It is clarified above that when ‘school social capital’ and ‘neighborhood social capital’ are described, this is referring to the students’ individual perceptions of social capital in these settings. Therefore, the social capital variables in the current study are analyzed at the individual level. Fourth, socio-economic status has been reported by children and this is also a limitation in the current study. Fifth, there is a possibility of measurement errors in school social capital since the adolescents filled the questionnaires out during class. It is possible that adolescents could feel uncomfortable answering questions about school. Additionally, clustering adolescents into groups in accordance with their scores on items increased the possibility of type 1 error. Since our target population was adolescents, future studies should investigate the relationships of all three social capital determinants with self-rated health among college students, adults, and the elderly. Also, it is necessary to conduct a longitudinal study to detect possible causality between social capital and self-rated health among the above-mentioned target groups.

## Conclusion

In general, several key findings should be highlighted. In the present study, self-rated health was positively related to family support, neighborhood trust, and vertical school trust when variables were entered separately into the model. When variables were entered simultaneously, family support and neighborhood trust remained significant predictors for self-rated health among adolescents. Mutual support and understanding within cohesive families, along with a healthy neighborhood, serve as a protective factor for children’s and youths’ health. In the future, an action plan needs to be incorporated within the educational system for tracking how social capital is developing among children and between children and their families and neighborhood.

## Paper context

Social capital has been widely studied in the context of health behavior in adult populations, but research on the impact of social capital on self-rated health in adolescents has been lacking scientific proof. In this paper, we report on the impact of different contexts and types of social capital on the self-rated health of Lithuanian adolescents. This nationally representative research indicates that self-rated health is positively associated with family, neighborhood, and school social capital. Therefore, we can conclude that mutual support serves as a protective factor for children’s and youth’s health. Policies for improving social capital for children are needed.
